# A Combretastatin-Mediated Decrease in Neutrophil Concentration in Peripheral Blood and the Impact on the Anti-Tumor Activity of This Drug in Two Different Murine Tumor Models

**DOI:** 10.1371/journal.pone.0110091

**Published:** 2014-10-09

**Authors:** Anja Bille Bohn, Thomas Wittenborn, Anne Sofie Brems-Eskildsen, Tinne Laurberg, Lotte Bonde Bertelsen, Thomas Nielsen, Hans Stødkilde-Jørgensen, Bjarne Kuno Møller, Michael R. Horsman

**Affiliations:** 1 Department of Experimental Clinical Oncology, Aarhus University Hospital, Aarhus, Denmark; 2 Department of Pathology, Aarhus University Hospital, Aarhus, Denmark; 3 The MR-Research Centre, Aarhus University Hospital, Aarhus, Denmark; 4 Department of Clinical Immunology, Aarhus University Hospital, Aarhus, Denmark; Witten/ Herdecke University, Germany

## Abstract

The vascular disrupting agent combretastatin A-4 disodium phosphate (CA4P) induces fluctuations in peripheral blood neutrophil concentration. Because neutrophils have the potential to induce both vascular damage and angiogenesis we analyzed neutrophil involvement in the anti-tumoral effects of CA4P in C3H mammary carcinomas in CDF1 mice and in SCCVII squamous cell carcinomas in C3H/HeN mice. Flow cytometry analyses of peripheral blood before and up to 144 h after CA4P administration (25 and 250 mg/kg) revealed a decrease 1 h after treatment, followed by an early (3–6 h) and a late (>72 h) increase in the granulocyte concentration. We suggest that the early increase (3–6 h) in granulocyte concentration was caused by the initial decrease at 1 h and found that the late increase was associated with tumor size, and hence independent of CA4P. No alterations in neutrophil infiltration into the C3H tumor after CA4P treatment (25 and 250 mg/kg) were found. Correspondingly, neutrophil depletion in vivo, using an anti-neutrophil antibody, followed by CA4P treatment (25 mg/kg) did not increase the necrotic fraction in C3H tumors significantly. However, by increasing the CA4P dose to 250 mg/kg we found a significant increase of 359% in necrotic fraction when compared to neutrophil-depleted mice; in mice with no neutrophil depletion CA4P induced an 89% change indicating that the presence of neutrophils reduced the effect of CA4P. In contrast, neither CA4P nor 1A8 affected the necrotic fraction in the SCCVII tumors significantly. Hence, we suggest that the initial decrease in granulocyte concentration was caused by non-tumor-specific recruitment of neutrophils and that neutrophils may attenuate CA4P-mediated anti-tumor effect in some tumor models.

## Introduction

Vascular disrupting agents are drugs that target the existing vasculature in tumors [1], [2]. These drugs induce a transient or permanent vascular shutdown which eventually leads to increased tumor necrosis [3]–[5]. Tubulin-binding agents are a subgroup of the VDAs and include the drug Combretastatin A-4 disodium phosphate (CA4P) [1], [6]. Upon tubulin binding, CA4P induces morphological changes in endothelial cells, which leads to vasoconstriction and decreased blood flow [3], [4], [7]–[10]. CA4P has been shown pre-clinically to have moderate to extensive effects on reducing tumor perfusion, increasing tumor necrosis, and inhibiting tumor growth [4], [5], [11]–[14], but the exact mechanisms for these effects are not fully understood. Additional studies have demonstrated that CA4P can significantly enhance tumor response to more conventional therapies, such as radiation and chemotherapy [3], [11], [12], [15], [16]. Clinically, CA4P has undergone testing both as a solitary agent [17]–[19] and in combination with other therapies [20], [21]. One of those clinical studies reported an increase in the number of granulocytes in peripheral blood 4 and 6 h after CA4P treatment [19], but the significance of this effect is not clear. In vitro studies by Westlin and colleagues showed that neutrophil derived proteases mediate disruption of the endothelial monolayer [22]. In contrast, Yang and co-workers showed that granulocytes derived from tumor bearing mice promote angiogenesis, reduce tumor necrosis, and enhance tumor growth by regulating bioavailability of VEGF [23]. Consistent with both scenarios a recent study in mice further confirmed that neutrophils present in tumors are capable of being either pro- or anti- tumorigenic dependent on the tumor microenvironment [24]. Since neutrophils have the potential to both induce vascular damage and angiogenesis, they possess the ability to indirectly either support or oppose the anti-tumoral effect of CA4P treatment. The aim of the current study was to investigate the potential of CA4P to modify neutrophil levels in the peripheral blood of mice over a 144-hour period following drug injection. Since our previous studies had demonstrated that CA4P not only induces effects in tumors [12], [13], but also resulted in physiological changes in non-tumor bearing animals [25], [26] we studied the neutrophil changes in mice with and without tumors. Finally, we also determined whether the observed changes could have any influence on the anti-tumor activity of CA4P.

## Materials and Methods

### Ethics Statement

All experiments were conducted in accordance with National and International guidelines and the protocol was approved by the Danish Animal Experiments Inspectorate’s approval (J.nr.2010/561-1919, C5). All efforts were made to minimize suffering.

### Animals and tumor models

Male CDF1 mice were obtained from Taconic Laboratories (Ry, Denmark) and male C3H/HeN mice were obtained from Harlan UK Ltd (Bicester, UK). Foot tumors were established in 10-14-week-old mice by injecting C3H mammary carcinoma cells into the right rear foot of CDF1 mice or SCCVII squamous cell carcinoma cells into the right rear foot of C3H/HeN mice. The C3H mammary carcinoma is an anaplastic adenocarcinoma that arose spontaneously in C3H mice at our institute and was originally designated as HB [27]; the name was changed to C3H mammary carcinoma when it was grown in the more stable CDF1 mouse variant [28]. The SCCVII originally arose in the abdominal wall of C3H mice in Dr. Herman Suit’s laboratory at Harvard University, Boston, Massachusetts, and was subsequently transferred to Karen Fu’s laboratory at the University of California in San Francisco and then to Dr. Robert Kallman’s laboratory at Stanford University in California [29]. Dr. Michael Horsman finally brought it to our department when he moved from Stanford University. Both tumor models are standard models used in our laboratory and the derivation and maintenance have previously been described [30], [31]. Tumor-bearing mice were included in experiments 10–20 days after inoculation, when tumors reached a size of about 150–250 mm^3^. Tumor volume was calculated from the formula D1 * D2 * D3 * π/6, where the D values represent the three orthogonal diameters.

### Combretastatin preparation and injection

Combretastatin A-4 disodium phosphate (CA4P) was supplied by OXiGENE, Inc. (San Francisco, CA). The drug was prepared fresh before each experiment, by dissolving in saline, and then kept cold and protected from light until used. CA4P was injected intraperitoneally (i.p.) at a dose of either 25 or 250 mg/kg (injection volume was 0.02 ml/g body weight for both doses). As control for CA4P animals were injected with drug solvent (saline) with an injection volume of 0.02 ml/g body weight.

### Blood samples and tumor excision

In one experiment 4 groups were included: 1) non-tumor bearing CDF1 mice treated with 25 mg/kg CA4P, 2) non-tumor bearing CDF1 mice treated with 250 mg/kg CA4P, 3) C3H tumor bearing CDF1 mice treated with 25 mg/kg CA4P, 4) C3H tumor bearing CDF1 mice treated with 250 mg/kg CA4P. In each of these 4 groups blood samples were withdrawn from the sub-orbital sinus 0, 1, 3, 6, 24, 48, 72, 96, 120, or 144 hours after CA4P injection. We used vehicle-treated mice as control groups. Blood samples from these groups were withdrawn 1 h after injection but were depicted in the graphs at t = 0 h to be able to distinguish these from blood samples withdrawn 1 hour after CA4P treatment. To avoid repetitive blood sampling from animals with small blood volumes only one blood sample was drawn from each mouse (n = 5–15). The blood samples were drawn directly into dry EDTA-tubes (Saratedt, Nümbrecht, Germany) and analyzed by flow cytometry as described below. Mice were killed by cervical dislocation and the tumors were excised and divided; one part was stored in liquid nitrogen and used for luminex analysis of cytokines (described below) and the other part was fixed in 4% formaldehyde and used for immunohistochemestry (described below). In a separate experiment non-tumor bearing C3H/HeN mice were included. These mice were treated with CA4P (25 mg/kg) or saline (control) and blood samples were taken 1 h after treatment as described above (n = 5).

### CA4P effect on granulocyte concentration

Whole blood was transferred to TRU COUNT tubes (BD Bioscience, Broendby, Denmark) or falcon tubes (BD Biosciences), depending on the experiment. The tubes were incubated for 15 minutes at room temperature with FITC conjugated anti-Gr-1 (Cedarlane, Ontario, Canada) antibodies to detect granulocytes. Red blood cells were lysed using Stock solution×10 (Ampliqon Bioreagents and molecular diagnostics, Skovlunde, Denmark) and the samples were analyzed by flow cytometry (FACS Calibur or FACSCanto II, BD Biosciences) and Flow Jo (version 9.3.1, Tree Star Inc., Ashland, OR). The anti Gr-1 antibody binds to Ly-6G, which is expressed by neutrophils, and to Ly-6C, which is expressed by neutrophils, dendritic cells, and subpopulations of lymphocytes and monocytes [32]. Granulocytes were therefore identified based on localization in a dot plot of forward and side scatter and by being Gr-1 positive.

### CA4P effect on the number of dead or apoptotic granulocytes

Blood samples were taken from the sub-orbital sinus of three CDF1 mice directly into dry EDTA tubes. Each blood sample was divided and used for all four groups included in the study: 1) CA4P, 2) CA4, 3) DMSO, and 4) saline. To 100 µl whole blood 2 µl of CA4P, CA4, or saline were added (the final concentrations of CA4P and CA4 were 10 µM). Because a single drop of DMSO is required to dissolve CA4 we included group 3, where 2 µl of a solution containing one drop of DMSO in 5 ml saline was added to 100 µl whole blood. All samples were incubated for two hours at 37°C. The numbers of dead and/or apoptotic granulocytes in each group were determined using flow cytometry as described above using Annexin V (BD Pharmingen, Broendby, Denmark) and BD Via-Probe (BD Biosciences).

### Estimation of neutrophil granulocyte area fraction in histological sections

Tumors fixed in 4% formaldehyde (described above), were embedded in paraffin and tissue sections of 2–3 µm were cut. For each tumor two sections, 200 µm apart, were produced. Endogenous peroxidases were blocked with H_2_O_2_, followed by thorough rinsing in PBS-TEG buffer, pH 9.0. The sections were then transferred to a LabVision Autostainer 480 (Labvision, Fremont, USA) and incubated overnight with rabbit anti-myeloperoxidase polyclonal antibody diluted 1∶200 (Nordic BioSite, Copenhagen, Denmark) or the negative control rabbit IgG (DAKOCytomation, Glostrup, Denmark). The primary antibody was detected by incubating sections for 30 min. with anti-rabbit IgG-horseradish peroxidase-conjugated polymers (DakoCytomation). Finally, sections were counterstained with Mayers hematoxylin and pictures of the sections were scanned using a NanoZoomer 2.0-HT (Hamamatsu Photonics K.K., Hamamatsu city, Japan). In order to estimate the degree of neutrophil granulocyte infiltration, we estimated the area fraction of neutrophils in the tumor by point-counting at ×20 magnification. The area fractions were estimated using a counting frame (240×240 µm, consisting of 100 equidistant-spaced points). Frames were sampled systematically throughout each tumor section, and an average (± SEM) of 38±1 frames were counted per tumor section. Points hitting necrosis, artifacts, or cells from normal non-neoplastic connective-tissue were ignored and so were neutrophils located in these areas. A neutrophil cell profile was defined as a cell with myeloperoxidase positive granules in the cytoplasm located in close proximity to a nucleus. The area fraction was calculated by dividing the number of points hitting a neutrophil granulocyte by the number of points hitting tumor tissue.

### Quantification of tumor cytokine level after CA4P treatment

Tumor tissue frozen in liquid nitrogen was weighed and transferred to sample tubes (RB tubes, Qiagen, Hilden, Germany) containing a stainless steel bead (Qiagen) and 20 µl buffer/mg tissue were added. The buffer consisted of NP40 cell lysis buffer (Invitrogen, Taastrup, Denmark), proteaseinhibitor cocktail (SIGMA-ALDRICH, Broendby, Denmark), and phenylmethylsulfonyl fluoride (PMSF) (SIGMA-ALDRICH). The samples were homogenized twice for 2 min. at 20 Hz and 4°C using a TissueLyser II (Qiagen), centrifuged for 10 min at 20,817 g at 4°C, and the supernatants were transferred to clean tubes. Prior to luminex analysis the total protein concentration in each sample was determined by adding 50 µl Bio-Rad protein assay (Bio-Rad Laboratories, Inc, Copenhagen, Denmark) and analyzing on a spectrometer Ceres UV900HDi (Bio-Tek instruments, inc., Winooski, VT). The level of chemokine (C-X-C motif) ligand 1 (KC/CXCL1), macrophage inhibitory protein 1α (MIP-1α/CCL3), macrophage inhibitory protein 2 (MIP-2/CXCL-2), and vascular endothelial growth factor (VEGF) in control tumors and tumors excised 1, 3, and 6 h after CA4P treatment were determined using a Milliplex map Kit according to the manufactures instructions (Millipore Corporation, Billerica, MA).

### Neutrophil depletion

The antibody 1A8 (Nordic BioSite), which binds specifically to Ly-6G [32], an antigen expressed by neutrophils, was used to deplete neutrophils in vivo. In a pilot study, we found that dilution in PBS and a dose of 25 µg 1A8 was optimal. 1A8 was diluted in PBS immediately before administration to a concentration of 50 µg/ml and 500 µl was injected i.p. As a control for 1A8, 500 µl PBS was i.p injected. In one experiment we included 4 groups of non-tumor bearing CDF1 mice: 1) PBS, 2) 1A8, 3) CA4P (25 mg/kg), and 4) 1A8+CA4P (25 mg/kg). In each of these groups, blood samples were taken from the sub-orbital sinus 0, 24, 48, or 72 h after injection (n = 5). Only one blood sample was taken from each mouse to avoid repetitive blood sampling from animal with small blood volumes. In a second experiment, we included 4 similar groups of C3H/HeN mice. Blood samples in this study, however, were only taken 24 h after injection (n = 10).

### Effect of neutrophil depletion on CA4P-mediated necrotic fraction estimates

Tumor bearing mice were divided into six groups (n = 6–21). Mice were injected with 1A8 or with PBS as a control for the antibody. Twenty-four hours later (t = 0) the mice were treated with CA4P (25 mg/kg or 250 mg/kg) or with saline, as a control for CA4P. Forty-eight hours after the first injection (t = 24 h) mice were sacrificed by cervical dislocation and tumors were excised, fixed in formalin, embedded in paraffin, and cut in sections of 2–3 µm. For each tumor a randomly selected section was cut and two additional, equally spaced sections (400 µm apart) were produced. The sections were hematoxylin and eosin stained and were microscopically examined using a ×6.3 objective. Each section was systematically scanned and projected on a grid with 60 equidistant-spaced points. For each field of vision, the total number of points overlying tumor (nT) and necrosis (nN) were recorded, and necrotic fraction of the tumor was defined by ΣnN/ΣnT.

### Statistics

Non-gaussian data were presented as median values with the 25^th^ and 75^th^ percentile as error bars and all other data were presented as mean value ± SEM unless stated otherwise. Data were compared using two-tailed Student’s t-test for pair wise comparison, one way ANOVA or Kruskal-Wallis One Way Analysis of Variance on Ranks followed by Dunn’s multiple comparisons of each group versus control groups where applicable for comparison of more than two groups. Necrotic fractions were compared and the interaction between the CA4P-mediated effect and neutrophil depletion was analyzed using a two way ANOVA for both tumor models with CA4P treatment and neutrophil depletion as the two independent variables. For all tests p<0.05 was considered statistically significant.

## Results

### CA4P affects the granulocyte concentration in peripheral blood

**Figure 1:**
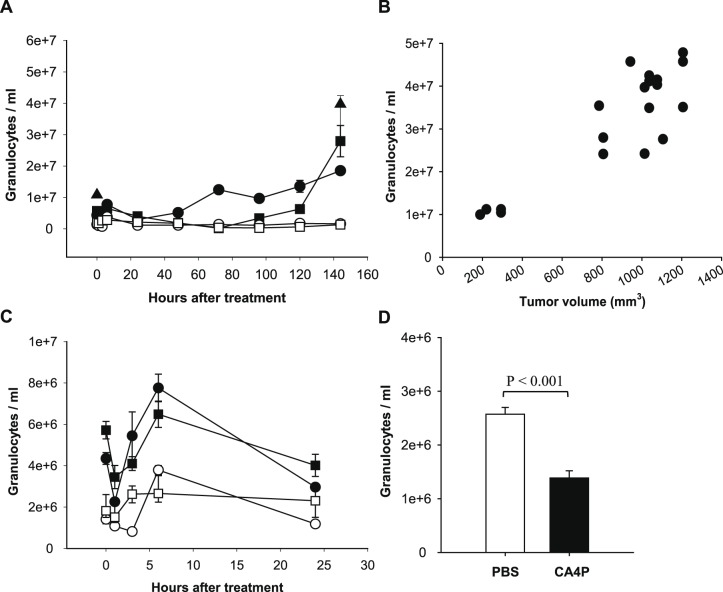
Granulocyte concentration in peripheral blood after CA4P treatment. A) and C) Granulocytes/ml peripheral blood as a function of hours after treatment with 25 mg/kg (• ○) or 250 mg/kg (▪ □) CA4P in C3H mammary carcinoma bearing (• ▪) and non-tumor bearing (○ □) CDF1 mice (n = 5–15). Blood samples from the saline-treated groups are depicted in the graphs at t = 0 h. In A) ▴ shows the granulocyte concentration in tumor bearing mice 1 h (depicted at t = 0) and 144 h after a saline injection (n = 15). Kruskal-Wallis One Way Analysis of Variance on Ranks was used to analyze for changes over time within each CA4P dose compared to t = 0 in each group. C) is a close up of the first 24 hours after treatment (shown in A). B) Granulocyte concentration as a function of tumor volume in CDF1 mice (n = 14). Pearson’s correlation coefficient = 0.88, p<0.001. D) Granulocytes/ml peripheral blood 1 h after treatment with PBS or 25 mg/kg CA4P in SCCVII squamous cell carcinoma bearing C3H/HeN mice (n = 10, Student’s t-test p<0.001). Data is presented as median values and the 25^th^ and 75^th^ percentiles (error bars) in A) and C), as single values in B), and as mean ± SEM in D).

To ascertain the effect of CA4P on the granulocyte level in peripheral blood over time, we treated both tumor bearing and non-tumor bearing CDF1 mice with either 25 or 250 mg/kg CA4P (4 groups in total). We withdrew one blood sample from each mouse at various time points after treatment, and used saline treated mice as reference (t = 0 hours in figure 1A). Using a Kruskal-Wallis One Way Analysis of Variance on Ranks to compare changes over time we found a significant increase in the number of granulocytes in tumor-bearing mice 72 h after treatment (CA4P dose of 25 mg/kg) 144 h (CA4P dose of 250 mg/kg) after treatment compared to t = 0 in each group (figure 1A). To determine whether the increased granulocyte concentration at these late time points was caused by the presence of the tumor, we investigated the granulocyte concentration 144 h after injection of saline. We found that this concentration was significantly increased compared to a blood sample drawn 1 hour after saline injection (See figure 1A; Student’s t-test p<0.05) and that it was at the level of the CA4P treated tumor-bearing groups at t = 144 h. The granulocyte concentration in the saline-treated group at depicted at t = 0 in this experiment is higher than in the other saline treated groups depicted at t = 0. This may be due to the fact that we used a different flow cytometer, with different threshold options, in this experiment. As shown in figure 1B, the granulocyte concentration increased as tumors grew (Pearson’s correlation coefficient r = 0.88, p<0.001). Thus, despite the high level of granulocytes in peripheral blood at t = 144 h, these numbers were not significantly different from the granulocyte number in saline treated tumor-bearing mice at this time point.

Looking at the first 24 hours of the experiment (figure 1C), it was clear that the level of granulocytes in peripheral blood was higher in saline-treated tumor-bearing mice (t = 0 h) than in the saline-treated non-tumor-bearing mice (t = 0 h; Student’s t-test: p<0.01). Furthermore, an oscillating change in the number of granulocytes in the tumor bearing mice was detected. We observed a significant decrease in granulocyte number 1 h after treatment compared to the saline-treated group (Kruskal-Wallis One Way Analysis of Variance on Ranks: p<0.001 and p = 0.003 for 25 and 250 mg/kg, respectively), followed by an increase 6 h after treatment (Kruskal-Wallis One Way Analysis of Variance on Ranks: p<0.001 for 25 mg/kg compared to the saline treated group). At later time points, the number of granulocytes returned to the level of the saline-treated mice (figure 1C). In the non-tumor bearing mice we found similar changes after both 25 and 250 mg/kg CA4P although they were less pronounced and at a slightly different time course. To investigate whether this CA4P induced phenomenon was specific for CDF1 mice, we included a group of C3H/HeN mice bearing a SCCVII squamous cell carcinoma (figure 1D). Similar to what we found in the CDF1 mice, we observed a significant decrease in the granulocyte concentration 1 h after treatment with 25 mg/kg CA4P compared to the granulocyte concentration in mice treated with PBS (Student’s t-test: p<0.001).

### CA4P effect on the number of dead or apoptotic granulocytes

**Figure 2:**
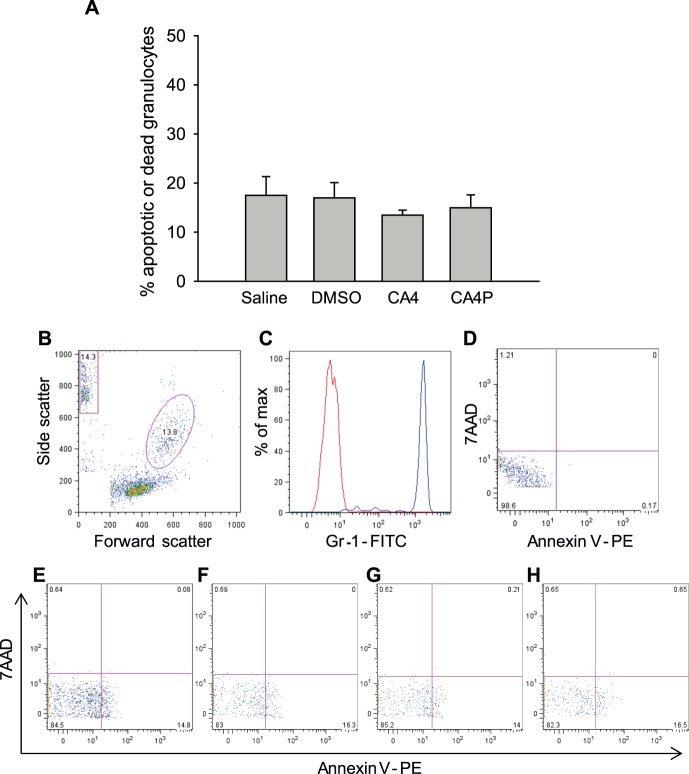
In vitro CA4P toxicity assay. A) *In vitro* effect of 2 h incubation of whole blood in the presence of CA4P (10 µM), CA4 (10 µM), DMSO diluted in saline, or saline on the percentage of apoptotic or dead granulocytes. Data is presented as mean + SEM (n = 3). B) A representative forward-side scatter demonstrating gating of the granulocytes (the pink oval gate). C) Verification of Gr-1 positivity of cells in the granulocyte gate. The red line shows the isotype control and the blue line represents the granulocytes. D–H) Annexin V (expressed by apoptotic cells) and 7AAD (expressed by dead cells) expression in granulocytes from D) a negative control (no 7AAD or annexin V staining), or after incubation with E) saline, F) DMSO, G) CA4, or H) CA4P.

To investigate whether the drop in granulocyte concentration 1 h after treatment was caused by CA4P toxicity towards granulocytes we incubated whole blood in the presence of saline, CA4P, the active drug CA4, or a drop of DMSO dissolved in saline. After 2 h of incubation the percentage of dead and/or apoptotic granulocytes was determined using flow cytometry. As shown in figure 2, we observed no significant changes in the percentage of apoptotic and/or dead granulocytes in any of the groups compared to saline-treated cells.

### Estimation of neutrophil granulocyte area fraction in histological sections

**Figure 3:**
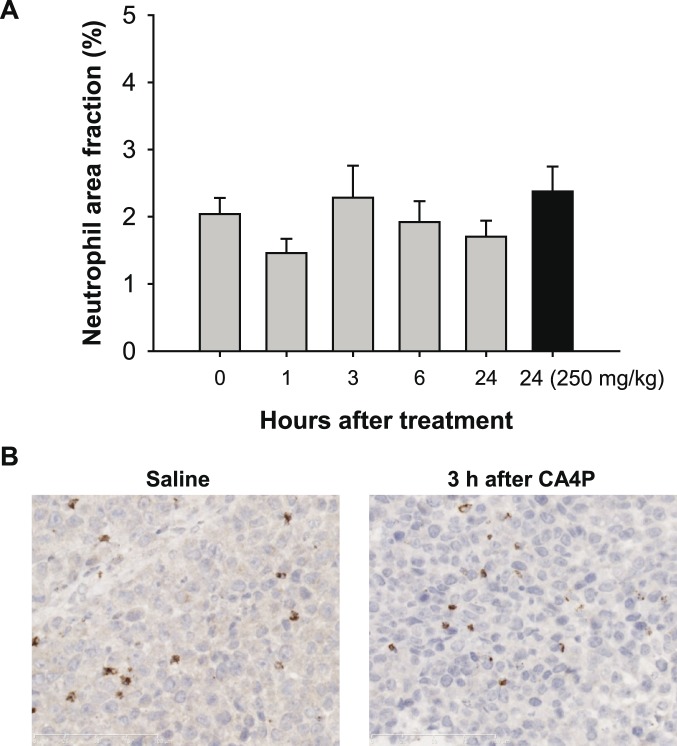
Estimated area fraction of myeloperoxidase positive granulocytes in tumor tissue. A) The estimated area fraction of myeloperoxidase positive granulocytes in tumor tissue from C3H mammary carcinoma bearing CDF1 mice treated with 25 mg/kg CA4P for 1, 3, 6, or 24 hours, with 250 mg/kg CA4P for 24 hours, or in mice injected with saline. B) Representative tumor tissue sections from mice injected with saline and mice treated with 25 mg/kg CA4P (3 hours post-injection). The sections have been stained with anti-myeloperoxidase antibody before estimation. Data is presented as mean + SEM (n = 4–9).

Since CA4P was non-toxic to neutrophils we analyzed whether neutrophils entered the tumor after CA4P treatment. Figure 3 shows the estimated area fraction of myeloperoxidase positive neutrophil granulocytes in tumors from saline-treated mice or 1, 3, 6, or 24 h after treatment with 25 mg/kg CA4P and 24 h after treatment with 250 mg/kg CA4P. CA4P treatment did not influence the area fraction of neutrophil granulocytes in tumor tissue at any of the investigated time points.

### Quantification of tumor cytokine level after CA4P treatment

**Figure 4:**
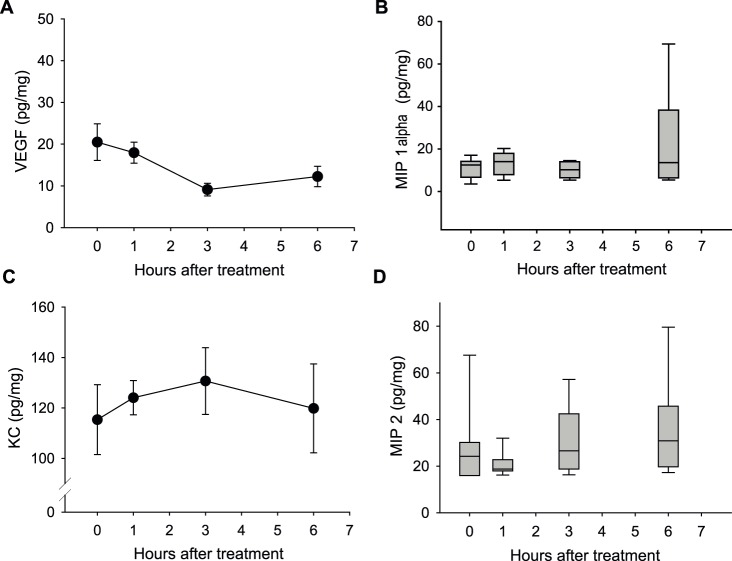
The effect of CA4P treatment on cytokine levels in C3H mammary carcinomas in CDF1 mice. A) VEGF, B) MIP-1α (CCL3), C) KC (CXCL1), and D) MIP-2 (CXCL2) concentration in tumors as a function of hours after treatment with 25 mg/kg CA4P and in tumors from saline-treated mice. A) and C) Data is presented as mean ± SEM. B) and D) Data is presented as the median (horizontal bar), the 25^th^ and 75^th^ percentile (bottom and top of boxes) and the 10^th^ and 90^th^ percentiles (error bars). For all data n = 10.

Neutrophils are recruited by cytokines such as KC (CXCL1), MIP-2 (CXCL2) and MIP-1α (CCL3) [33], [34] and once the neutrophils are there, they are capable of secreting VEGF [35], a cytokine that can induce angiogenesis [36]. Hence, we analyzed the level of these four cytokines in tumors from mice treated with saline and in tumors excised 1, 3, and 6 hours after treatment with 25 mg/kg CA4P (figure 4). Three hours after CA4P treatment the level of VEGF in tumors was lower than the level in saline-treated tumors (figure 4A). This difference was, however, not significant (p = 0.069). We found no difference in KC (CXCL1), MIP-2 (CXCL2) and MIP-1α (CCL3) levels (figure 4B, C, and D) between saline and CA4P treated tumors either.

### Depletion of neutrophils

**Figure 5:**
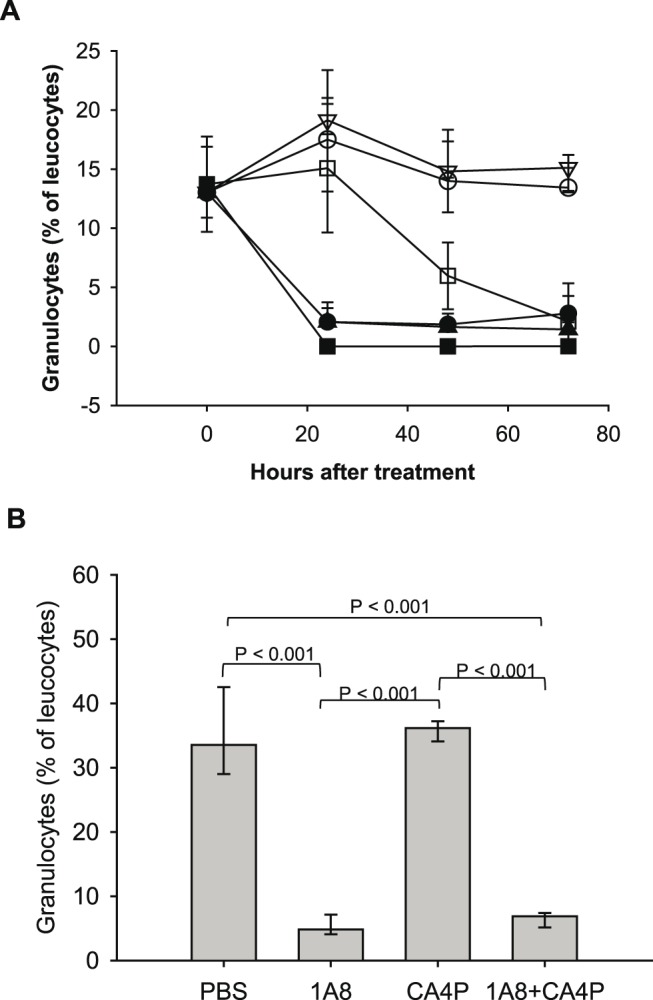
Verification of neutrophil-depletion in CDF1 and C3H/HeN mice. A) Granulocytes (percentage of leucocytes) in non-tumor bearing CDF1 mice as a function of hours after treatment (n = 5). The mice were treated with PBS (○), the anti-neutrophil antibody 1A8 (•), CA4P (25 mg/kg, ▿), 1A8 and CA4P (25 mg/kg) in combination (▴), CA4P (250 mg/kg, □), or 1A8 and CA4P (250 mg/kg) in combination (▪). B) Granulocytes as a percentage of leucocytes in C3H/HeN mice 24 h after injection of PBS, 1A8, CA4P (25 mg/kg), or 1A8 and CA4P in combination (n = 5). The data is presented as median values and error bars represent the 25^th^ and 75^th^ percentiles.

We depleted neutrophils in vivo in non-tumor bearing CDF1 mice by injecting the 1A8 antibody which is specific for Ly-6G expressed by neutrophils and which has been shown to deplete neutrophils in mice [32]. After 24 hours, the neutrophil number in 1A8-treated CDF1 mice was significantly reduced to less than 20% when compared to mice injected with PBS (figure 5A, p = 0.008) and to less than 30% in the C3H/HeN mice (figure 5B, p = 0.016). Although CA4P treatment (250 mg/kg) on its own reduced the number of circulating neutrophils, CA4P did not influence the 1A8-mediated neutrophil depletion at neither 25 nor 250 mg/kg.

### Effect of neutrophil depletion on CA4P-mediated necrotic fraction estimates

We analyzed the effect of neutrophil depletion on CA4P-mediated anti-tumor activity by comparing necrotic fraction in mice treated with 25 mg/kg or 250 mg/kg CA4P with or without neutrophil depletion beforehand (table 1). In the C3H mammary carcinoma we found that the necrotic fraction in tumors of CA4P treated animals was higher than the necrotic fractions in tumors from mice treated with PBS (p = 0.06 and p<0.001 for 25 and 250 mg/kg, respectively). After neutrophil depletion the CA4P-mediated necrotic fraction was significantly increased when compared to the 1A8 group (p<0.001 at both doses). The CA4P-mediated change in necrotic fraction was larger in neutrophil depleted mice (table 1) than in mice with no neutrophil depletion (64% versus 28% at 25 mg/kg and 359% versus 88% at 250 mg/kg). In the SCCVII sqaumous cell carcinoma neither CA4P nor 1A8 treatment changed the necrotic fraction significantly compared to the control group (p>0.5 for all groups). Only in the C3H mammary carcinoma using 250 mg/kg CA4P the difference between the CA4P-mediated effects in neutrophil- and non-neutrophil-depleted animals where actually statistically significant (p<0.001).

**Table 1:** Effect of neutrophil depletion on CA4P-mediated necrosis.

Tumor	Treatment	Necrotic fraction	Change (%)
**C3H**	Control	19.8±1.4	
	CA4P (25 mg/kg)	25.4±2.6	28
	CA4P (250 mg/kg)	37.4±3.4*	89
	1A8	13.8±1.1	
	1A8+CA4P (25 mg/kg)	22.7±1.7*′	64
	1A8+CA4P (250 mg/kg)	63.4±2.2*′	359
**SCCVII**	Control	3.0±0.4	
	CA4P (25 mg/kg)	2.7±0.8	−10
	CA4P (250 mg/kg)	4.5±0.4	50
	1A8	5.3±1.5	
	1A8+CA4P (25 mg/kg)	1.5±0.3	−72
	1A8+CA4P (250 mg/kg)	6.6±2.2	24

1A8: anti-neutrophil antibody, CA4P: Combretastatin A-4 disodium phosphate.

*signifcantly different from the control group,

*′significantly different from the 1A8 group. Values are mean ± SEM (n = 5–21).

## Discussion

In the present study, we determined the effect of CA4P treatment on the granulocyte concentration in peripheral blood in two different mice strains. We confirm a previous study in humans reporting an increase in the number of granulocytes in peripheral blood 4 and 6 hours after CA4P treatment [19], but we also report a significant decrease in neutrophil concentration 1 hour after treatment, a time interval not investigated in that clinical study. The effect of the changes in neutrophil concentration on the anti-tumor effect of CA4P treatment is, to our knowledge, not known. Neutrophils theoretically have the potential to influence the effect of CA4P treatment either by mediating damage to the vasculature [37] or by inducing angiogenic progression [23]. Thus, the aim of this study was to investigate the role of neutrophils in the CA4P-mediated effect.

We analyzed the level of granulocytes in peripheral blood in non-tumor bearing and C3H mammary carcinoma bearing CDF1 mice and found that the granulocyte concentration in the tumor bearing mice was higher than the concentration observed in non-tumor bearing mice. Moreover, a massive increase in the granulocyte concentration in the tumor bearing mice 144 hours after both CA4P and saline treatment was observed. We thus concluded that the changes observed at the late time points were caused by the tumor, and hence independent of CA4P treatment. At the early time points, we observed a decrease in granulocyte number 1 hour after treatment followed by an increase exceeding the granulocyte levels of saline-treated mice (t = 0). We found a similar, and statistically significant, effect 1 hour after CA4P treatment in C3H/HeN mice. This decrease in granulocyte concentration could be caused by CA4P being toxic to neutrophil granulocytes or by recruitment of these cells to CA4P-mediated damage in tumor vessels [4], [7], [11] to either increase or repair the damage. In vitro results showed that the drug was non-toxic to neutrophil granulocytes. Hence, with the purpose of investigating recruitment of neutrophils to tumors, we histologically estimated the number of these cells in non-necrotic tumor areas of C3H tumors from mice treated with CA4P. We were not able to demonstrate any difference between the neutrophil granulocyte area fractions in tumors from CA4P treated mice and saline-treated mice at any of the time-points or CA4P doses investigated. Parkins et al. [38] and Welford et al [39] have previously shown that CA4P mediates an increase in the number of tumor infiltrating neutrophils 18 and 24 h after treatment, respectively. Their observations were made histologically using myeloperoxidase (like us) and an anti-Gr1 antibody, respectively but in different tumor models. Similarly, a study in a murine colon cancer model showed an increase in tumor associated neutrophils 24 hours after treatment with 5,6-Dimethylxanthenone-4-acetic acid (DMXAA), another vascular disrupting agent [40]. In contrast to these studies, we did not include neutrophil granulocytes present in necrotic areas in the data presented here, because this would rather be an indication of the degree of necrosis.

Our result was supported by the fact that we did not observe any CA4P-induced changes in three chemokines involved in recruitment of neutrophils (KC, MIP-2, and MIP-1α) [33], [34]. The plasma concentration of KC (and other cytokines) has previously been shown to increase in both tumor bearing and non-tumor bearing mice 4 hours after treatment with CA4P [41]. These CA4P-mediated changes in cytokine expression were therefore not induced as a result of CA4P effects in the tumor and thus, they support our observation of no significant changes in KC concentration in the tumor. In addition, we did not observe any significant changes in the angiogenesis-inducing cytokine VEGF [36], [42]. VEGF can be released by tumor cells and different host cells (i.e., non-tumor cells) [43] including neutrophils [35] and we would therefore not be able to conclude anything based on changes in this cytokine level alone. But, a change in neutrophil granulocyte area fraction and a similar change in VEGF level would point us in a direction as to whether neutrophils indirectly could affect the efficacy of CA4P.

Despite the above-mentioned observations we assumed that neutrophils could still be recruited to sites of injury in damaged vessels, mediating their positive or negative action there. We therefore depleted neutrophils in CDF1 mice and C3H/HeN mice using the 1A8 neutrophil antibody which binds to Ly-6G, an antigen expressed by neutrophils. This treatment reduced the number of granulocytes to 20%–30% of what we observed in control CDF1 or C3H/HeN mice. However, this neutrophil depletion appeared to have different effects in the two tumor models. After neutrophil depletion, CA4P treatment induced a larger necrotic fraction in the C3H tumor compared to mice without neutrophil depletion at both 25 and 250 mg/kg suggesting that in the presence of neutrophils the actual effect of CA4P treatment was reduced. In contrast, in the SCCVII tumor neither CA4P treatment alone or in combination with neutrophil depletion induced any significant changes in necrotic fraction compared to control tumors. A two way analysis of variance demonstrated that neutrophil depletion only affected the CA4P-mediated necrotic fraction significantly in the C3H tumor and only after treatment with the high dose of CA4P (250 mg/kg). The fact that tumor associated neutrophils can diminish the effect of CA4P is in accordance with other studies, in different tumor models, which have demonstrated that neutrophils play a role in tumor growth and especially tumor angiogenesis [24], [44], [45].

We did not observe neutrophil recruitment to the tumors after CA4P treatment although we detected changes in neutrophil concentration in peripheral blood in both tumor bearing and non-tumor bearing mice after CA4P treatment. A recent study by Taylor et al. [46] analyzed the effect of CA4P treatment on circulating endothelial progenitor cell (CEP) concentration in peripheral blood. Similar to our study, they reported two peaks in the CEP concentration after treatment, one early and one late. These peaks occur at approximately the same time points as the changes in granulocyte concentration that we found. However, the increase that we observed at the late time points did not seem to peak but rather continued increasing consistent with our observation that granulocyte concentration increased as tumors grew, with or without CA4P treatment. The peak in CEP and granulocyte concentration at the early time point may be caused by the same effect. Taylor et al. argue that the early peak in CEP concentration does not occur as a response to or a consequence of CA4P treatment and like us, they observed this early peak in both tumor bearing and non-tumor bearing mice. Neutrophil depletion changed the necrotic fraction in our study albeit only in one of the two included tumor models and at a high CA4P dose. It is possible that the decrease in granulocyte concentration observed in this study was caused by non-specific recruitment of granulocytes to tissues throughout the mice body. Despite the fact that CA4P is tumor specific [4], [7], [11] blood flow/perfusion changes have been observed in normal tissue [5], [13] albeit to a lesser extent than observed in tumors. Hence, it is possible that vessels in normal tissue experience small changes and although it does not cause vascular damage, it may be sufficient to recruit neutrophils. We therefore suggest that early drop in granulocyte number observed 1 hour after CA4P injection was caused by neutrophil recruitment to vessels throughout the body. As a consequence, an abundant load of neutrophils may be released from the bone marrow and spleen into the peripheral blood in order to compensate for the loss of granulocytes, which results in the peak observed at 3–6 hours after treatment. Although we did not show an increased recruitment of neutrophils at any of the investigated time points our data showed that the presence of neutrophils may attenuate CA4P-mediated anti-tumor effects when CA4P is used at high doses in some tumors.
